# Biophysical assay for tethered signaling reactions reveals tether-controlled activity for the phosphatase SHP-1

**DOI:** 10.1126/sciadv.1601692

**Published:** 2017-03-24

**Authors:** Jesse Goyette, Citlali Solis Salas, Nicola Coker-Gordon, Marcus Bridge, Samuel A. Isaacson, Jun Allard, Omer Dushek

**Affiliations:** 1 Sir William Dunn School of Pathology, University of Oxford, Oxford, U.K.; 2 Wolfson Centre for Mathematical Biology, University of Oxford, Oxford, U.K.; 3 Department of Mathematics and Statistics, Boston University, Boston, MA 02215, USA.; 4 Department of Mathematics, University of California, Irvine, Irvine, CA 92697, USA.

**Keywords:** tethered signalling, clustered receptors, allosteric activation, surface plasmon resonance, enzymatic catalysis, tyrosine phosphatase, SHP-1, mathematical model, stochastic simulations, Biochemistry

## Abstract

Tethered enzymatic reactions are ubiquitous in signaling networks but are poorly understood. A previously unreported mathematical analysis is established for tethered signaling reactions in surface plasmon resonance (SPR). Applying the method to the phosphatase SHP-1 interacting with a phosphorylated tether corresponding to an immune receptor cytoplasmic tail provides five biophysical/biochemical constants from a single SPR experiment: two binding rates, two catalytic rates, and a reach parameter. Tether binding increases the activity of SHP-1 by 900-fold through a binding-induced allosteric activation (20-fold) and a more significant increase in local substrate concentration (45-fold). The reach parameter indicates that this local substrate concentration is exquisitely sensitive to receptor clustering. We further show that truncation of the tether leads not only to a lower reach but also to lower binding and catalysis. This work establishes a new framework for studying tethered signaling processes and highlights the tether as a control parameter in clustered receptor signaling.

## Teaser

Tethering of the cytoplasmic tyrosine phosphatase SHP-1 to clustered receptors increases its activity 900-fold.

## INTRODUCTION

A common theme in signal transduction pathways is the tethering of signaling enzymes near their substrates before catalysis (*1*, *2*). Familiar examples include reactions on surface receptors, where cytoplasmic enzymes first bind to receptor tails (tethers) before catalyzing reactions on substrates within reach. Understanding of these complicated reactions is limited because they depend not only on the catalytic rate but also on the tether reach and on the binding kinetics that localize the enzyme. Moreover, many cell surface receptors cluster, but how clustering influences reaction rates is poorly understood (*3*).

A large group of immune surface receptors rely on the tethering of cytoplasmic kinases and phosphatases to both initiate and integrate signaling (*4*). Their unstructured cytoplasmic tails contain multiple tyrosines that serve as both docking sites and substrates for these enzymes. In the case of inhibitory immune receptors (for example, PD-1 and LAIR-1), tyrosines in conserved immunotyrosine-based inhibitory or switch motifs (ITIMs or ITSMs) generate docking sites for the SH2 domains of the cytosolic phosphatases SHP-1 and/or SHP-2. When tethered, these phosphatases are thought to undergo allosteric catalytic activation (*5*–*8*) to dephosphorylate various membrane-proximal tyrosines (*9*–*11*).

Microscopy studies have highlighted the clustering of immune receptors on the plasma membrane (*11*–*15*), but the consequences of clustering remain poorly defined. For example, it is presently unknown how membrane localization and the degree of clustering influence the local substrate concentration experienced by SHP-1. Mathematical models can predict large local substrate concentrations for certain tethers (*16*–*18*), which may even override the catalytic specificity of enzymes (*19*). This may explain the observation that SHP-1 and SHP-2 can regulate the phosphorylation state of the clustered inhibitory receptors they interact with (*11*).

**Fig. 1:**
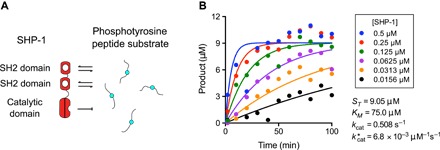
Solution-based assays coarse-grain reaction mechanisms for the tyrosine phosphatase SHP-1. (**A**) Schematic of the domain structure of SHP-1 and a subset of reactions that may occur in solution with peptide substrates. (**B**) Standard solution–based enzymatic assay showing the production of inorganic phosphate (product) over time for the indicated concentration of SHP-1 mixed with the PEG12-ITIM substrate (data are representative of two independent experiments). Progress curves are fit with a mathematical model to provide an estimate for $k_{\text{cat}}^{*}$ (see Materials and Methods).

Solution-based in vitro assays for enzymatic activity have been instrumental to our understanding of signaling and, particularly, to SHP-1 (*5*, *6*). These experiments measure the reaction product over time after mixing the enzyme and substrate in solution, and model fitting produces an estimate for the overall catalytic rate $k_{\text{cat}}^{*}$ (= *k*_cat_/*K*_m_, where *K*_m_ is the Michaelis constant) (*20*). Applying this assay to SHP-1 (Fig. 1, A and B) makes it clear that this single number coarse-grains the reaction mechanism when proteins have multiple domains that interact with substrates, and moreover, the tether does not influence these reaction rates.

Surface plasmon resonance (SPR), as implemented in commercial instruments such as Biacore (GE Healthcare), is a widely used biophysical assay for molecular interactions (*21*). In a typical experiment, one binding partner is immobilized to a surface, whereas the other is injected over it. The instrument reports a highly accurate measure of the mass of material bound at the surface, expressed as resonance units (RU), as a function of time. The resulting data are fit to mathematical models to determine the association rate (*k*_on_) and dissociation rate (*k*_off_). High sensitivity and accuracy and the availability of many surface chemistries resulted in the method gaining considerable popularity not only for biomedical research but also for medical diagnostics, food safety and security, and environmental monitoring (*21*). Despite these advances, the method remains largely a tool for the study of molecular binding.

Here, we retool SPR for the study of tethered enzymatic reactions applied to SHP-1. Injection of SHP-1 over a surface immobilized with phosphorylated peptides produced a noncanonical SPR trace as a result of tethered dephosphorylation reactions of clustered peptides. Using a mathematical analysis that captures both the spatial and stochastic elements of tethered reactions, we show that five biophysical and biochemical constants can be independently extracted from a single SPR trace. We found that binding of either of the SH2 domains to the tether allosterically activated SHP-1 and that the tether length modulated not only the reach but also the binding and catalysis. Using these parameters, we find that tethering increases reaction rates by 900-fold with a tether-induced local increase in substrate concentration as the dominant contribution, but only when receptors are clustered within 5 nm. Collectively, this work highlights tethering as a control parameter for signaling reactions and provides a previously unreported SPR-based platform for the study of tethered signaling, with implications for drug discovery.

## RESULTS

### Tethered enzymatic reactions produce a noncanonical SPR trace

**Fig. 2:**
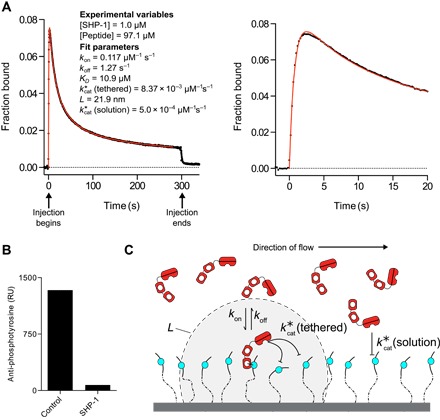
An SPR assay recovers five independent biophysical/biochemical constants characterizing tethered enzymatic reactions by SHP-1. (**A**) Representative SPR trace (black dots) for SHP-1 injected over a surface immobilized with 48.5 RU of an ITIM peptide derived from LAIR-1 on a 28-repeat PEG linker (PEG28-ITIM). A fit of the MPDPDE model (red line) provides estimates for the indicated parameters. Early time data and fit are shown on the right. Unprocessed data are shown in fig. S1. (**B**) Anti-phosphotyrosine antibody injected at the end of the experiment shows reduced binding in the experimental flow cell (SHP-1) compared to a buffer-injected flow cell with equivalent peptide levels (control). (**C**) Schematic of reactions taking place when SHP-1 is injected over a surface of immobilized phosphorylated peptides. Note that peptide anchoring is displayed in one dimension for clarity, but because peptides are randomly coupled to a dextran matrix, which extends 100 to 200 nm above the surface, they are anchored in three dimensions.

To create a substrate surface for SHP-1 in SPR, we coated a surface with peptides containing an ITIM sequence, from the N terminus of the inhibitory receptor LAIR-1, with a 28-repeat polyethylene glycol (PEG) spacer (PEG28-ITIM; Table 1). When SHP-1 was injected over this surface, we observed a complicated curve with an initial binding phase that was quickly followed by a reduction in binding despite continuous injection of SHP-1 (Fig. 2A). To convert the arbitrary response units reported by the SPR instrument to a more meaningful unit, we normalized this curve to maximum binding, assuming a one-to-one interaction with peptide (see Materials and Methods). We confirmed that SHP-1 was dephosphorylating the substrate by injecting an anti-phosphotyrosine antibody following the injection of SHP-1, which revealed near complete dephosphorylation (Fig. 2B).

**Table 1:** Peptides used in study (phosphotyrosines are denoted as Y*).

**Name**	**Sequence**	**Contour length**
PEG28-ITIM	Biotin-(PEG)_28_-DLQEVTY*IQLDHH	12.1 nm
PEG12-ITIM	Biotin-(PEG)_12_-DLQEVTY*IQLDHH	6.5 nm
PEG3-ITIM	Biotin-(PEG)_3_-DLQEVTY*IQLDHH	3.3 nm
PEG0-ITIM	Biotin-GDLQEVTY*IQLDHH	2.7 nm

The decrease in binding, despite continuous injection of SHP-1, can be understood by considering the catalytic activity of the enzyme that over time destroys binding sites for its SH2 domains. When SHP-1 is first injected over the surface, it begins binding via the SH2 domains (initial rise within the first 3 s; Fig. 2A). This binding (or tethering) increases the dephosphorylation rate by confining SHP-1 and its phosphorylated substrates to a restricted volume, resulting in the rapid destruction of highly clustered phosphorylated peptides (steep fall between 3 and 100 s; Fig. 2A). However, the rate of dephosphorylation by tethered SHP-1 decreases over time because the tethered enzyme is unable to reach remaining phosphorylated peptides, whose average distance increases over time. This inefficient tethered dephosphorylation combined with inefficient solution dephosphorylation leads to a slow loss in overall binding at later time points (slowly decreasing asymptote after 100 s; Fig. 2A). Consistent with this interpretation, we observed negligible binding but partial dephosphorylation when point mutations were introduced to both SH2 domains (fig. S2). These interactions are summarized in Fig. 2C.

### A mathematical model quantifies the tethered enzymatic SPR assay

The tethered enzymatic SPR assay is heavily influenced by stochastic fluctuations. This may seem counterintuitive because the instrument reports macroscopic binding averaged over picomoles of protein across a millimeter-scale surface. However, tethered catalytic reactions are limited to the number of peptide substrates within reach, which we estimate to be ~8 initially (assuming [peptide] = 100 μM with a reach of 25 nm) and over time to reach 0. Therefore, the SPR trace represents the average of many realizations of a low copy number stochastic process.

**Fig. 3:**
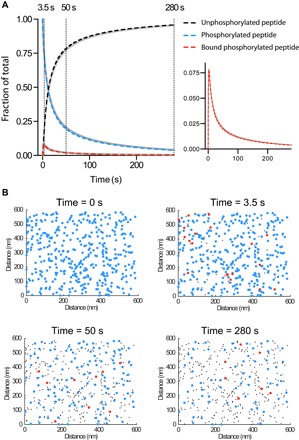
Mathematical models capture the physics and chemistry in tethered enzymatic SPR. (**A**) Levels of unphosphorylated peptide, phosphorylated peptide, and phosphorylated peptide bound to SHP-1 over time determined using the stochastic simulation (solid gray lines) or the MPDPDE model (dashed colored lines). Levels of SHP-1 bound to phosphorylated peptide are replotted for clarity (right panel). Good agreement is observed between the stochastic simulation and the MPDPDE model calculation. (**B**) Snapshots of the spatial distribution of the three molecular species at indicated time points from the stochastic simulation [colors as in (A)]. Initially, the phosphorylated peptides are randomly distributed on the surface (0 s), but as time progresses, clustered peptides are effectively dephosphorylated by tethered catalysis (50 s), ultimately resulting in phosphorylated peptides too far apart for efficient tethered catalysis (280 s). These two-dimensional spatial distributions are generated from the stochastic simulation by projecting 20 nm in the third dimension. See Materials and Methods for computational details. Parameters: [SHP-1] = 1 μM, [peptide] = 100 μM, *k*_on_ = 0.1 μM^−1^ s^−1^, *k*_off_ = 1 s^−1^, $k_{\text{cat}}^{*}(\text{tethered})$ = 0.01 μM^−1^ s^−1^, *L* = 20 nm, and $k_{\text{cat}}^{*}(\text{solution})$ = 0.0005 μM^−1^ s^−1^.

We therefore developed a spatial stochastic simulation to reproduce the tethered enzymatic SPR assay. The model includes the kinetics of SHP-1 binding to phosphorylated peptides by its SH2 domains [governed by the on-rate (*k*_on_) and off-rate (*k*_off_) constants], the dephosphorylation of peptides when SHP-1 is bound to the surface [$k_{\text{cat}}^{*}(\text{tethered})$], or when SHP-1 is free in solution [$k_{\text{cat}}^{*}(\text{solution})$] (Fig. 2C). The local concentration of peptide experienced by tethered SHP-1 is determined by the reach parameter *L*, which is defined as the quadrature average of the average reach distance of the peptide and the average reach distance of SHP-1 bound to a peptide. This calculation is based on approximating the motion of both the free and bound peptides using the worm-like chain polymer model (see Materials and Methods). The stochastic simulation was used to plot the three molecular species over time and to provide spatial snapshots of these species at different times (Fig. 3). As expected, we found that clustered peptides were preferentially destroyed, leading to a nonrandom distribution of the surviving phosphorylated peptides.

Stochastic simulations often provide intuition but are not practical for data fitting because they require long computation times. We therefore developed a computationally efficient model. Standard deterministic ordinary differential equation (ODE) models fail to fit the experimental data because they do not account for stochastic fluctuations (fig. S3). We therefore used the multicenter particle density (MPD) formalism, previously used to study defects in solid-state physics (*22*–*24*), to develop a hybrid integral MPD partial differential equation (MPDPDE) model that includes the reactions specified for the stochastic simulation (see Materials and Methods). We found an agreement between the stochastic simulation and the computationally efficient MPDPDE model (Fig. 3A).

We used the MPDPDE model to examine the dependency of the predicted SPR trace on the experimental variables (SHP-1 and peptide concentrations) and on the five model parameters [*k*_on_, *k*_off_, $k_{\text{cat}}^{*}(\text{tethered})$, *L*, and $k_{\text{cat}}^{*}(\text{solution})$]. We found that the binding trace shifted in nonintuitive ways (fig. S4). For example, changing the concentration of peptide, which in standard SPR simply changes the scale of the binding trace, resulted in a change to the shape of the binding trace because a different proportion of peptides was dephosphorylated by tethered versus solution enzyme. This underlines the need for a mathematical analysis of the data.

We next analyzed the SPR data using the MPDPDE model. We found an excellent fit of the model to the data (Fig. 2A, red line) and recovered the five model parameters. We performed Markov chain Monte Carlo (MCMC) analysis to determine whether a different set of parameters can produce the same binding trace, but we found that the five recovered parameters are unique (fig. S5). In summary, the computationally efficient MPDPDE model captures the stochastic fluctuations in tethered reactions and can recover five parameters from a single SPR trace.

### Fitted biophysical and biochemical constants are independent of experimental variables

**Fig. 4:**
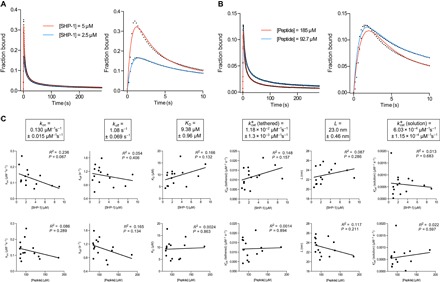
Fitted biophysical/biochemical parameters are independent of experimental variables. Representative SPR traces (black dots) and MPDPDE model fits (solid color) for (**A**) two SHP-1 concentrations and (**B**) two initial peptide concentrations with the right panels showing early time data and fit. (**C**) Plots of fitted parameters versus SHP-1 concentration (upper row) and peptide concentration (lower row) with linear regression fits (*R*^2^ and *P* values are indicated) reveal a lack of correlation, indicating that the fitted parameters do not depend on the experimental variables. Averages of fit parameters with SEMs from all experiments are shown in boxes (*n* **=** 15). All experiments are performed using wild-type SHP-1 and phosphorylated PEG28-ITIM peptides. Exclusion criteria for experiments exhibiting long time scale artifacts, such as nonspecific binding and/or differential flow cell drift, are discussed in Materials and Methods (Quality control) and fig. S6.

A key test of a mathematical model is the ability to recover the same parameter values when different experimental variables are used. This is particularly important for SPR, where mass transport and rebinding can produce parameters that are dependent on the concentration of surface-immobilized receptors (*25*, *26*). We therefore performed experiments at different SHP-1 concentrations and immobilized peptide concentrations (Fig. 4, A and B). The fitted parameters did not correlate with either experimental variable and, moreover, showed excellent reproducibility (Fig. 4C). As predicted by the model, changing the concentration of immobilized peptide led to a change in the shape of the SPR binding trace (Fig. 4B), highlighting the difficulty of interpreting the data without performing model fitting.

### Fitted biophysical and biochemical constants are consistent with the biology of SHP-1

The recovered parameters (Fig. 4C) were within the expected range for tethered signaling by SHP-1. The affinity of SHP-1 interacting with the LAIR-1 ITIM (*K*_D_ = 9.38 μM) is in agreement with that for isolated SH2 domains of SHP-1 interacting with other ITIMs (*27*). We observed a 20-fold increase in the tethered over solution catalytic rate [$k_{\text{cat}}^{*}(\text{tethered})$ = 1.18 × 10^−2^ μM^−1^ s^−1^ versus $k_{\text{cat}}^{*}(\text{solution})$ = 6.03 × 10^−4^ μM^−1^ s^−1^], which is consistent with an allosteric activation of SHP-1 when bound by SH2 domains (*6*). We note that the standard solution–based assay recovered an overall catalytic rate that was between these two rates ($k_{\text{cat}}^{*}$ = 6.8 × 10^−3^ μM^−1^ s^−1^; Fig. 1B), consistent with a combination of inactive and allosterically active SHP-1 mediating dephosphorylation in solution.

The tethered enzymatic SPR assay also produced an estimate for the reach parameter (*L*), which was 23 nm. This number corresponds to a phosphorylated substrate experiencing a maximum local SHP-1 concentration of 45 μM when tethered, compared to, for example, a concentration of 1 μM in solution (Fig. 2A) or in the cytoplasm of immune cells (see Discussion). To further appreciate the effect of surface tethering, we used the fitted parameters to calculate the time required to dephosphorylate 50% of the peptides with (9.2 s) and without tethering (19 min), revealing that tethering reduced the reaction time by 125-fold (fig. S7).

### The tether length controls the binding, catalysis, and reach parameters

**Fig. 5:**
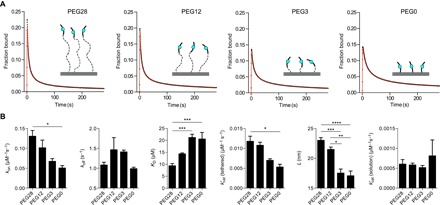
Reduction in tether lengths reduces the reach parameter and introduces configurational steric hindrance reducing *k*_on_ and $k_{\mathrm{cat}}^{*}(\text{tethered})$. (**A**) SPR traces (black circles) and MPDPDE model fits (red lines) of SHP-1 injected over peptides with 28 (PEG28), 12 (PEG12), 3 (PEG3), and 0 (PEG0) PEG linker repeats. (**B**) Average fit parameters for PEG28-ITIM (n = 15), PEG12-ITIM (n = 3), PEG3-ITIM (n = 3), and PEG0-ITIM (n = 2) show reduced values of L, *k*_on_, and $k_{\text{cat}}^{*}(\text{tethered})$ for shorter linkers. Two-way analysis of variance (ANOVA) with a Bonferroni multiple comparison correction is used to determine the P values (****P < 0.0001, ***P < 0.001, **P < 0.01, *P < 0.05).

To further understand the effects of the reach parameter (*L*), we performed experiments with different tether lengths. To do this, we reduced the length of the spacer from 28 to 0 PEG repeats without modifications to the peptide (Table 1). The model produced excellent fits to all data (Fig. 5A), and as expected, the reach parameter decreased with decreasing tether lengths (Fig. 5B). Although there is a large difference in the contour length between the longest (PEG28, 12.1 nm) and shortest (PEG0, 2.3 nm) tethers, no marked decrease in *L* (23 nm for PEG28 and 17 nm for PEG0) was observed. This implied that SHP-1 itself contributes significantly to the reach length when it is tethered. This can be understood by noting that, although the contour length of the tethers may be long, the average reach distance is relatively short due to the small persistence length of flexible PEG (*28*) and polypeptides (*29*). Thus, the rigid domains of SHP-1 may contribute much more to the reach length than one might intuitively expect.

We note that a decrease in *k*_on_ and $k_{\text{cat}}^{*}(\text{tethered})$ was also observed (Fig. 5B), which likely reflects steric hindrance at short tether lengths (PEG3 and PEG0). This form of configurational hindrance is a result of a smaller fraction of time that short linkers spend sufficiently far from their anchor point to accommodate SHP-1 binding. This mechanism is not expected to change *k*_off_, which is consistent with the similar *k*_off_ values we find across tethers. This effect is significant and indicates that SPR experiments to measure binding affinities should use long linkers to avoid configurational steric hindrance.

### A different reach but a similar allosteric activation is induced by each SH2 domain of SHP-1

**Fig. 6:**
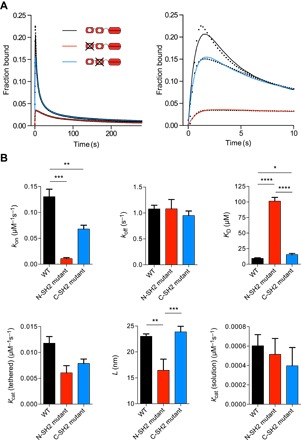
Binding by either SH2 domain allosterically activates SHP-1, but the reach parameter is larger for N-SH2 binding. (**A**) SPR traces (black dots) and MPDPDE model fits (solid lines) of the N- and C-terminal SH2 domain binding–null mutants and wild-type SHP-1 injected over PEG28-ITIM. (**B**) Average fit parameters for wild-type (WT) SHP-1 (*n* = 15), N-terminal SH2 (N-SH2) mutant SHP-1 (*n* = 3), and C-terminal SH2 (C-SH2) mutant SHP-1 (*n* = 9) show weak binding and reduced reach when SHP-1 binds via the C-terminal SH2 domain compared to the N-terminal SH2 domain, but allosteric activation is observed in both cases. Two-way ANOVA with a Bonferroni multiple comparison correction is used to determine the *P* values (****P < 0.0001, ***P < 0.001, **P < 0.01, *P < 0.05).

Given that SHP-1 itself may significantly contribute to the reach length, we hypothesized that binding by the N-terminal SH2 domain would allow SHP-1 to reach further compared to the C-terminal SH2 domain. We generated SHP-1 variants with inactivating point mutations to either SH2 domain. Mutation of the N-terminal SH2 domain showed drastically reduced binding, whereas mutation of the C-terminal SH2 domain showed a weak effect on binding (Fig. 6, A and B), clearly demonstrating that the N-terminal SH2 domain dominates the interaction of wild-type SHP-1 to the membrane-proximal ITIM of LAIR-1.

As expected, a reduction in the reach parameter was observed for the N-terminal mutant (*L* = 16.5 nm) compared to the wild-type (*L* = 23.0 nm) and C-terminal mutant (*L* = 23.9 nm) because binding via the C-terminal SH2 reduced the overall reach (Fig. 6B). This difference is more than twice as large as the spatial extent of the C-terminal SH2 domain (~3 nm, estimated from structure), which reflects the large effective persistence length of structured domains.

In contrast to previous studies, we found that the N-terminal mutant still exhibited allosteric activation because $k_{\text{cat}}^{*}(\text{tethered})$ remained 10-fold larger than $k_{\text{cat}}^{*}(\text{solution})$. Therefore, binding of either the N- or C-terminal SH2 domain is sufficient to allosterically activate SHP-1. We also found that the *k*_on_ differed by ~10-fold between the N- and C-terminal SH2 domains, but the *k*_off_ was nearly identical (Fig. 6B).

## DISCUSSION

Our understanding of tethered signaling reactions is limited by the lack of experimental methods. We have described a previously unreported SPR-based assay for tethered enzymatic reactions that, from a single experiment, can recover five biophysical and biochemical constants that quantify tethered signaling for SHP-1 with clustered substrates. We demonstrate that these constants can be determined with high accuracy, as a result of the high sensitivity of SPR, and we further show that they are independent of the SHP-1 and substrate concentrations.

**Fig. 7:**
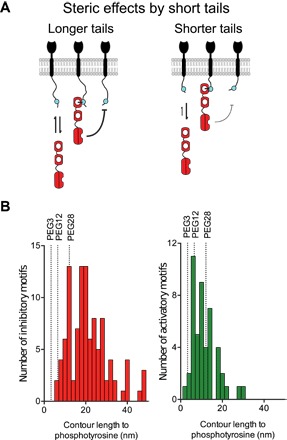
Steric penalty to binding and catalysis by short tethers. (**A**) Shorter cytoplasmic tails (tethers) result in decreased *k*_on_ for binding and $k_{\text{cat}}^{*}(\text{tethered})$ for catalysis as a result of steric hindrance (indicated by thickness of black arrow). (**B**) Histograms of the contour length (number of amino acids **×** 0.3 nm per amino acid) to phosphotyrosine residues on inhibitory receptors (ITIM/ITSM; mean length of 21.2 nm, red histogram) and activatory receptors (ITAM/ITTM/YxxM; mean length of 11.3 nm, green histogram) for human receptors. Most inhibitory receptor tails are longer than PEG28 where steric penalties are lower, whereas most activatory receptor tails are shorter than PEG28 where steric penalties are higher.

We observed that reducing tether lengths below ~12 nm (PEG28) introduces a steric penalty to binding, implying a lower bound on the cytoplasmic tails of inhibitory receptors that recruit SHP-1 (Fig. 7A). A bioinformatic analysis of inhibitory receptors reveals that most receptors contain ITIMs that are located ≥12 nm from the plasma membrane (Fig. 7B). Most activatory receptors contain tyrosines that are located ≤12 nm from the plasma membrane. This finding raises the possibility that tethers may have a role in binding specificity by, for example, sterically preventing binding of signaling enzymes.

**Fig. 8:**
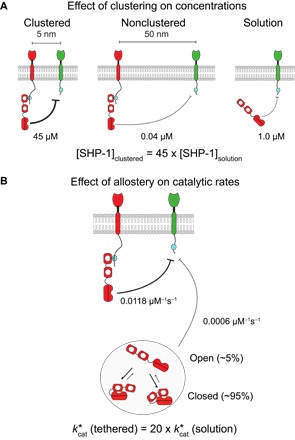
Clustering and allosteric activation increase the activity of tethered SHP-1 by 900-fold. (**A**) The concentration of SHP-1 experienced by the substrate when SHP-1 is tethered to clustered receptors (left), tethered to nonclustered receptors (center), and free in the cytoplasm (right). The concentration of SHP-1 increases 45-fold as it recruited from solution to clustered receptors. (**B**) The catalytic rate ($k_{\text{cat}}^{*}$) increases 20-fold when SHP-1 is tethered (bound) to a receptor compared to when it is in solution. This allosteric activation of SHP-1 upon binding is consistent with a dynamic transition between closed low-activity and open high-activity states while in solution. The combination of increased concentration (45-fold) and increased catalytic activity (20-fold) leads to a 900-fold increase in the overall dephosphorylation rate because SHP-1 is recruited from solution to clustered receptors.

Activatory and inhibitory immune receptors are both known to cluster in the plasma membrane (*11*–*15*), but the extent and consequences of clustering remain poorly understood. In the absence of clustering, a phosphorylation site on the cytoplasmic tails of these receptors will experience the low ~1-μM concentration of cytoplasmic SHP-1 [based on 280,000 copies of SHP-1 in cytotoxic T cells (*30*) with a radius of 5 μm]. Tethering of SHP-1 to nonclustered immune receptors at distances >50 nm results in even lower concentrations (for example, 0.04 μM when receptors are 50-nm apart), but when clustered within 5 nm, we can now estimate that this phosphorylated site will experience an SHP-1 concentration of 45 μM (Fig. 8A). This concentration is exquisitely sensitive to the degree of clustering so that a 10-fold decrease in receptor clustering (5 to 50 nm) results in a 1125-fold decrease in concentration (45 to 0.04 μM). These concentrations are calculated using the formula for σ with *L* = 23 nm for *r* = 5 and 50 nm (see Materials and Methods). We note that this is based on a reach of *L* = 23 nm, which represents SHP-1 bound to an ITIM on PEG28 dephosphorylating another ITIM on PEG28. We expect the value of *L* to decrease and, hence, the local concentration to increase when SHP-1 dephosphorylates other substrates such as ITAMs on shorter activatory receptor tails.

The current model for SHP-1 activation is based on an allosteric conformational change into an “open” high catalytic activity state induced by N-terminal SH2 domain binding (Fig. 8B) (*6*, *8*, *31*). In agreement with this model, we have found a 20-fold increase in the catalytic rate when the SH2 domains are engaged (tethered versus solution catalytic rates; Fig. 4C). The sensitivity of the present assay has revealed that the C-terminal SH2 domain can also induce the open state, suggesting that the “closed” low catalytic activity state may involve occlusion of the catalytic domain by either SH2 domain. The observation that unbound (solution) SHP-1 exhibits catalytic activity, albeit less efficiently, suggests that it is in equilibrium between closed and open states in solution. Assuming the open state transition is complete upon SH2 binding and that the activity of the open state is similar when tethered or when achieved spontaneously when unbound, our results suggest that SHP-1 spends only 5% of the time in the open active conformation in solution [that is, $k_{\text{cat}}^{*}(\text{solution})=0.05k_{\text{cat}}^{*}(\text{tethered})$].

The combined effects induced by SHP-1 tethering on allosteric activation (20-fold) and local substrate concentration (45-fold) can be summarized by calculating the dephosphorylation rate ([SHP-1] × $k_{\text{cat}}^{*}$). We find a similar rate when SHP-1 is in solution or tethered but not clustered (1 μM × 0.000603 μM^−1^ s^−1^ versus 0.04 μM × 0.0118 μM^−1^ s^−1^), but observe a 900-fold increase in the dephosphorylation rate when tethered and clustered (45 μM × 0.0118 μM^−1^ s^−1^).

These calculations and our SPR assay are likely to be valid for reactions within immune receptor clusters. However, SHP-1 and the highly homologous SHP-2 are involved in diverse reactions within cells that may include multivalent binding to diffusing receptors. Recent work using rule-based modeling for SHP-2 and phosphatidylinositol 3-kinase have highlighted the complex set of interactions that are possible with multivalent reactions across receptor tails and the importance of parameter values (*32*, *33*). Demonstrations of the enhanced avidity of SHP-1 and SHP-2 on bivalent substrates have largely been conducted on isolated tandem SH2 domains to eliminate dephosphorylation and simplify interpretation (*34*, *35*). Here, we found no evidence for bivalent reactions, which may reflect the ability of the protein tyrosine phosphatase (PTP) domain to dephosphorylate tyrosines before the C-terminal SH2 can bind. Future work with other substrates is needed to determine whether, and in what context, bivalent reactions can take place with an active PTP domain. Although the strength of our assay is the ability to simultaneously parse multiple parameters, mathematical models based on rule-based frameworks (*36*, *37*) are ultimately needed to translate the parameters we obtained into the diverse reactions taking place within cells.

To analyze the SPR data, we used an MPDPDE mathematical analysis that captures both the spatial and stochastic elements of tethered signaling. This analysis was previously developed to study annihilation reactions in solid-state physics (*22*–*24*). Standard ODE models based on first moment expansions fail to fit the data because, for example, they are unable to predict the formation of a nonhomogeneous distribution of phosphorylation produced by tethered reactions. Beyond the SPR assay, it is interesting to speculate that these tethered signaling reactions may lead to the appearance of large-scale phosphorylation patterns in cells. The appearance of spatial patterns of membrane-localized phosphorylation (*38*) and signaling enzymes (*39*, *40*) (often a proxy for phosphorylation) in T cells may be a result of tethered phosphorylation/dephosphorylation reactions. Future work in a reductionist setting can determine whether tethered reactions are sufficient to produce these patterns.

The biophysical assay for tethered enzymatic reactions introduced here can be used for the study of a large number of tethered signaling reactions on immune receptors (*4*). Although we have focused on the interactions with the tyrosine phosphatase SHP-1, the assay can be performed with a large number of tyrosine kinases, such as those of the Src and Syk families, which can both phosphorylate and bind their substrates. More generally, the method can be used in any situation where an enzyme can both bind and modify a substrate. Many such enzymes, SHP-1/SHP-2 included, are attractive therapeutic targets, and by providing rich mechanistic information, the assay may be particularly useful to identify drugs that target allosteric mechanisms (*41*) or tether components. Unlike the catalytic domains of the enzymes they recruit, tethers such as immune receptor cytoplasmic tails are often conserved in length but not in sequence, potentially allowing for more targeted therapeutics. The tethered enzymatic assay is a useful extension to the already widely used SPR platform for drug discovery and mechanistic studies (*21*), but we expect that it can be implemented in other instruments where binding can be observed over time (for example, Bio-Layer interferometry).

Tethered signaling reactions are complicated to study because they depend on multiple factors, such as binding kinetics, catalytic rates, allosteric activation, clustering, and tether length/flexibility. The SPR assay for tethered enzymatic reactions can parse these effects by providing five independent biophysical/biochemical parameters governing these reactions. When applied to SHP-1, the work has revealed that tethering increases enzymatic rates by 900-fold and that this increase is highly sensitive to the degree of receptor clustering. This work provides a new way to quantitatively study tethered signaling processes and has underlined the tether as a control parameter for signaling.

## MATERIALS AND METHODS

### Plasmids and peptides

A construct expressing murine SHP-1 with an N-terminal 6× His tag was provided by M. H. Brown. Mutation of the SH2 domains was performed using a quick-change strategy. The mutations introduced were R30K and R33E for the N-terminal SH2 domain mutant and R136K for the C-terminal SH2 domain mutant previously shown to result in loss of binding (*6*). All peptides were ordered from PeptideSynthetics and were certified to be >95% pure. Sequences of peptides used are shown in Table 1. Peptides sequences were derived from the membrane-proximal ITIM sequence of mouse LAIR-1 receptor.

### Protein production

SHP-1 DNA constructs were transformed into the BL21-CodonPlus (DE3)-RIPL strain (Agilent Technologies) *Escherichia coli* and plated on LB agar with ampicillin (100 μg/ml), and then grown overnight at 37°C. The next day, colonies were innoculated into a 10-ml LB selection medium [LB medium with ampicillin (100 μg/ml) and chloramphenicol (50 μg/ml)], grown overnight at 37°C, and then transferred to 1 liter of LB selection medium without chloramphenicol until the optical density at 600 nm was 0.6 to 0.8. The cells were then treated with isopropyl-β-d-thiogalactopyranoside (final concentration, 0.1 mM) and harvested by centrifugation after 20 hours of culture at 25°C.

Bacterial pellets were resuspended in tris-buffered saline [TBS; 20 mM tris(hydroxymethyl)aminomethane, 150 mM NaCl] with 0.5% Triton X-100 and protease inhibitors (protease inhibitor cocktail; Sigma), and then lysed with three 30-s bursts of sonication interspersed with 60-s rest periods on ice. Lysates were clarified by centriguation at 15,000 relative centrifugal force followed by filtration through a 0.45-μm filter. Clarified lysates were applied to the Ni^2+^-NTA resin, which was washed with 10 column volumes of TBS, followed by 10 column volumes of TBS with 30 mM imidazole, before SHP-1 protein was eluted with 50 mM imidazole in TBS (pH 7.5). Glycerol was added to a final concentration of 10% (v/v), and protein was stored in aliquots at −40°C until the day of experiment.

On the day of the experiment, aliquots of SHP-1 and mutants were thawed and further separated by size-exclusion chromatography and AKTA fast protein liquid chromatography (GE Healthcare Life Sciences) on a Superdex S75 10/300 GL column (GE Healthcare Life Sciences) equilibrated with 20 mM Hepes, 150 mM NaCl, 0.05% Tween 20, and 1 mM dithiothreitol. Concentrations of fractions containing SHP-1 were measured using the optical density at 280 nm, using a Nanodrop ND-1000 spectrophotometer (Thermo Scientific).

### Surface plasmon resonance

Experiments were performed on a Biacore 3000 instrument (GE Healthcare Life Sciences). All experiments were performed at 10°C and with a buffer flow rate of 10 μl/min. The buffer used was Hepes-buffered saline (HBS-EP; GE Healthcare Life Sciences), which contained 10 mM Hepes (pH 7.4), 150 mM NaCl, 3 mM EDTA, and 0.005% Surfactant P20.

A CM5 sensorchip was coupled with streptavidin to near saturation (typically between 4000 and 7000 RU) using the amine coupling kit (GE Healthcare Life Sciences) as described previously (*42*). After streptavidin was coupled, biotinylated peptides were injected to give the indicated concentrations in experimental flow cells, and excess biotin-binding sites were blocked with biotin in HBS-EP. The molar ratio of peptide to streptavidin was kept below 1:1 to ensure that peptide immobilization was random and not clustered on the tetravalent streptavidin molecules. Reference flow cells were treated with buffer and then blocked with biotin; pilot experiments using unphosphorylated control peptides in reference flow cells were indistinguishable from buffer-treated reference flow cells when injected with SHP-1. The chip surface was then conditioned with 5 × 5-min injections of HBS-EP. SHP-1 protein in HBS-EP with 1 mM dithiothreitol was then injected over reference and experimental flow cells in series for 5 min at the indicated concentrations. All SPR data were converted from the reference-subtracted data (in resonance units) to fraction bound by dividing the resonance units by the theoretical maximum resonance units expected if the experimental flow cell was saturated with bound SHP-1.

### Determination of peptide concentration

To determine the concentration of peptide in the assays, we first needed a conversion factor between the resonance units and the mass of peptide at the chip surface. We determined this conversion factor by injecting four concentrations of SHP-1 over a control flow cell on a CM5 chip and plotting the mass of SHP-1 injected against the raw resonance unit change. We repeated this on seven flow cells across four sensor chips to get an average slope of 149 ± 15 RU per g/liter (±SEM). This constant, together with the molecular weight of the peptide, was used to convert between the resonance units of peptides immobilized and the molar concentration at the chip surface. For example, 48.5 RU of PEG28-ITIM (molecular weight, 3221) was immobilized to obtain [peptide] = 97.1 μM (Fig. 2A).

### Quality control

From the MPDPDE model–simulated SPR traces, one would predict that the $k_{\text{cat}}^{*}(\text{solution})$ (fig. S4, G and H) and *L* (fig. S4F) are likely to be very sensitive to small systematic errors in the SPR trace at longer time scales. Nonspecific binding and baseline drift are two well-known sources of such systemic errors that can produce artifacts at long time scales (see fig. S6A for examples). To exclude data affected by these artifacts, we propose a simple quality control check that greatly improves the accuracy of estimating *L*, $k_{\text{cat}}^{*}(\text{tethered})$, and $k_{\text{cat}}^{*}(\text{solution})$. As a measure of the signal-to-noise ratio at long time points, we took the resonance units 20 s after the injection was completed (noise) and divided it by the resonance units 20 s before the injection finished (signal). We found that when the signal-to-noise ratio was greater than 20%, large aberrations in *L*, $k_{\text{cat}}^{*}(\text{tethered})$, or $k_{\text{cat}}^{*}(\text{solution})$ were apparent, depending on whether the drift was above or below baseline (fig. S6B). Data that had evidence of significant artifact were excluded from the study based on this criterion (red data points in fig. S6B).

### Calculation of local substrate concentration using a polymer model

A key component of the models (described below) is the calculation of the local substrate concentration that a tethered enzyme experiences. We assume that the motion of an unbound phosphorylated peptide (state *A*) and the motion of SHP-1 bound to a phosphorylated peptide (state *B*) can both be approximated by the worm-like chain model, which is a widely used polymer model. This model provides the probability of finding the tip of the polymer at position **r**

$$P(r)={\left(\frac{3}{2\pi l^{2}}\right)}^{3/2}\text{exp}(-\frac{3r\cdot r}{2l^{2}})$$

where $l=\sqrt{l_{c}l_{p}}$, with *l*_c_ as the contour length and *l*_p_ as the persistence length. When applied to the free phosphorylated substrate, this probability is taken to be the position of the phosphorylated tyrosine residue with *l* = *L*_*A*_. When applied to bound SHP-1, this probability is taken to be the position of the catalytic pocket of the phosphatase domain with *l* = *L*_*B*_. Using these probabilities, we can calculate the concentration of the substrate σ(*r*) that a tethered enzyme will experience when they are anchored a distance of *r* apart (fig. S8)

$$\sigma (r)=\int P_{A}(r\prime )P_{B}(r\prime -r)d^{3}r\prime$$

where the integration is over all space. Without loss of generality, we let $r=r\hat{z}$

$$\sigma (r)={\left(\frac{3}{2\pi L_{A}^{2}}\right)}^{3/2}{\left(\frac{3}{2\pi L_{B}^{2}}\right)}^{3/2}\int_{0}^{2\pi }d\varphi \int_{0}^{\pi }d\theta \int_{0}^{\infty }dr\prime {\left(r\prime \right)}^{2}\text{sin}(\theta )\times \;\text{exp}(-\frac{3{\left(r\prime \right)}^{2}}{2L_{A}^{2}})\text{exp}(-\frac{3}{2L_{B}^{2}}({\left(r\prime \right)}^{2}+{\left(r\right)}^{2}-2rr\prime \;\text{cos}(\theta )))$$

and by using the variable substitution $q={\left(r\prime \right)}^{2}+r^{2}-2rr\prime \;\text{cos}(\theta )$ and integrating over *q*, we find

$$\sigma (r)=(\frac{2\pi L_{B}^{2}}{3r}){\left(\frac{3}{2\pi L_{A}L_{B}}\right)}^{3}\int_{0}^{\infty }dr\prime r\prime \;\text{exp}(-\frac{3{\left(r\prime \right)}^{2}}{2L_{A}^{2}})\times (\text{exp}(-\frac{3}{2L_{B}^{2}}{\left(r\prime -r\right)}^{2})-\text{exp}(-\frac{3}{2L_{B}^{2}}{\left(r\prime +r\right)}^{2}))$$

Evaluating this integral and collecting terms leads to a simple analytical expression for the local substrate concentration

$$\sigma (r)={\left(\frac{3}{2\pi L^{2}}\right)}^{3/2}\text{exp}(-\frac{3r^{2}}{2L^{2}})$$

where $L=\sqrt{L_{A}^{2}+L_{B}^{2}}$ is the reach parameter.

We note that the parameter $L_{B}^{2}$ is the variance of the position of the tip of the polymer **r**, which for state *B* is a compound polymer composed of a phosphorylated peptide and SHP-1. This position can be decomposed into **r** = **r**_*A*_ + **r**_*S*_, where **r**_*A*_ is the position of the phosphorylated peptide and **r**_*S*_ is the position of SHP-1. Because we assume that the polymer is much longer than its persistence length, the random variables **r**_*A*_ and **r**_*S*_ are uncorrelated, and their variances sum linearly, leading to $L_{B}^{2}=L_{A}^{2}+L_{S}^{2}$, where *L*_*A*_ is a parameter associated only with the phosphorylated peptide and *L*_*S*_ is a SHP-1–specific parameter. Therefore, the reach parameter can be expressed as a function of the worm-like chain parameters for the phosphorylated peptide and SHP-1, $L=\sqrt{2L_{A}^{2}+L_{S}^{2}}$.

### Mathematical model reactions

We developed a stochastic and a deterministic model for tethered enzymatic SPR that are based on the same reactions. In this section, we describe the reactions in general before the models are described in the sections that follow.

The models are initialized with phosphorylated substrate distributed randomly in space (state *A*). A phosphorylated substrate can be bound by an enzyme (state *B*) with first-order kinetics

$$A\overset{k_{\text{on}}^{*}}{\underset{k_{\text{off}}}{⇌}}B$$

where $k_{\text{on}}^{*}$ and *k*_off_ are in units of s^−1^. The bimolecular on-rate (*k*_on_, in the unit μM^−1^ s^−1^) is related to the first-order on-rate by $k_{\text{on}}^{*}=k_{\text{on}}$ [SHP-1], where [SHP-1] is the concentration of the injected enzyme (in units of micromolar). When the enzyme is bound, it can dephosphorylate substrates within reach

$$A+B\overset{\mu \sigma (r;L)}{⇀}B+C$$

where *C* is the unphosphorylated substrate, σ(*r*) is the local concentration (in units of micromolar, see above), and μ is the surface catalytic rate (in the unit μM^−1^ s^−1^). We note that $\mu =k_{\text{cat}}^{*}(\text{tethered})$ and is used for clarity in the derivations of the mathematical models below. Last, phosphorylated substrate can be dephosphorylated by enzyme directly from solution

$$A\overset{\lambda }{⇀}C$$

where λ is the solution dephosphorylation rate (in the unit s^−1^). The solution catalytic rate, $k_{\text{cat}}^{*}(\text{solution})$, in the unit μM^−1^ s^−1^, is related to the solution dephosphorylation rate by $\lambda =k_{\text{cat}}^{*}(\text{solution})$ [SHP-1].

Note that *A*, *B*, and *C* represent peptide polymers that are anchored at a fixed location within the volume of the dextran matrix. We assume that the dextran matrix is stiff compared to the peptide polymers so that interactions between *A* and *B* in the matrix are determined primarily by the combined reach of the peptide polymers and the enzyme.

### Stochastic simulation

The overall state of the stochastic model can be represented by the positions of the substrate molecules and each molecule’s current chemical state (one of *A*, *B*, or *C*). Because the substrates are immobile, the system can be modeled by a collection of discrete-state jump Markov processes with rates (that is, propensities) for reactions as given in the preceding section. Our stochastic simulation engine generated exact realizations of these processes using the Gibson-Bruck next-reaction method (*43*) variant of the well-known stochastic simulation algorithm (*44*).

The simulation is initialized with a random distribution of peptide substrates in a cube. The side length of the cube is determined by the initial concentration of peptides and the absolute number of peptides, which is a simulation parameter taken to be 500,000. For computational efficiency, we define a maximum support of 4.5 × *L* so that reactions between a bound enzyme (*B*) and a free phosphorylated peptide substrate (*A*) that are anchored to a distance larger than the maximum support are ignored. This is reasonable because the concentration of substrate that a bound enzyme experiences at the maximum support is σ(4.5*L*) ≈ 10^− 14^ μM. Increasing the maximum support produced identical simulations but required longer computational times.

### Deterministic (standard) ODE calculations

A standard mean field model based on PDEs for tethered reactions leads to the following set of coupled equations

$$\frac{\partial A(r,t)}{\partial t}=-(k_{\text{on}}^{*}+\lambda )A(r,t)+k_{\text{off}}B(r,t)-\mu \int \sigma (r-r\prime )A(r,t)B(r\prime ,t)d^{3}r\prime \frac{\partial B(r,t)}{\partial t}=k_{\text{on}}^{*}A(r,t)-k_{\text{off}}B(r,t)$$

where *A* and *B* are functions of time (*t*) and space (**r**) with initial conditions *A*(**r**, *t* = 0) = *A*_*T*_ and *B*(**r**, *t* = 0) = 0. We note that, as a result of spatially homogeneous initial conditions, the solution will be spatially homogeneous at all times because there are no reactions that break spatial symmetry and, therefore, *A*(**r**, *t*) = *A*(*t*) and *B*(**r**, *t*) = *B*(*t*). Using these identities and rescaling by *A*_*T*_, we arrive at the following ODE system

$$\frac{\partial A(t)}{\partial t}=-(p_{1}+p_{4})A(t)+p_{2}B(t)-p_{3}A(t)B(t)\frac{\partial B(t)}{\partial t}=p_{1}A(t)-p_{2}B(t)$$

with *A*(*t* = 0) = 1 and *B*(*t* = 0) = 0, and the four fitting parameters (in the unit s^−1^) are related to the biophysical constants as follows: $p_{1}=k_{\text{on}}^{*}=k_{\text{on}}$ [SHP-1], p_2_ = *k*_off_, $p_{4}=\lambda =k_{\text{cat}}^{*}(\text{solution})$ [SHP-1], and

$$p_{3}=\mu A_{T}\int \sigma (r-r\prime )d^{3}r\prime =k_{\text{cat}}^{*}(\text{tethered})\times [\text{peptide}]$$

The expression of p_3_ highlights that this standard ODE model is independent of the reach parameter *L* because ∫σ(**r** − **r**′)*d*^3^**r**′ = 1. The value of *B*(*t*) was fit to experimental data using lsqcurvefit in Matlab (Mathworks) using the four fitting parameters (p_1_, p_2_, p_3_, and p_4_) but produced a poor fit (see fig. S3).

### Deterministic MPDPDE calculations

As discussed in the main text and shown in the previous section, a standard ODE model that does not account for stochastic fluctuations failed to fit the tethered enzymatic SPR data (fig. S3) and, moreover, did not agree with the stochastic simulations. The low copy number of substrates within reach of tethered enzymes means that stochastic effects are prevalent. To capture these effects, we used the MPD formalism previously used in the study of solid state physics (*22*, *23*).

We define $\rho_{m,m\prime }$ as the MPD

$$\rho_{m,m\prime }=\langle \prod_{i=1}^{m}n_{A}(r_{i},t)\prod_{j=1}^{m\prime }n_{B}(r_{j}^{\prime },t)\rangle$$

where variables in bold denote vector quantities. The explicit expression for the first five MPDs are

$$\begin{array}{c} \rho_{1,0}=\langle n_{A}(r_{1},t)\rangle \equiv n_{A}(t)\\ \rho_{0,1}=\langle n_{B}(r_{1}^{\prime },t)\rangle \equiv n_{B}(t)\\ \rho_{2,0}=\langle n_{A}(r_{1},t)n_{A}(r_{2},t)\rangle \equiv n_{A}^{2}(t)X_{A}(t,r_{1}-r_{2})\\ \rho_{0,2}=\langle n_{B}(r_{1}^{\prime },t)n_{B}(r_{2}^{\prime },t)\rangle \equiv n_{B}^{2}(t)X_{B}(t,r_{1}^{\prime }-r_{2}^{\prime })\\ \rho_{1,1}=\langle n_{A}(r_{1},t)n_{B}(r_{1}^{\prime },t)\rangle \equiv n_{A}(t)n_{B}(t)Y(t,r_{1}-r_{1}^{\prime }) \end{array}$$

where we have defined *n*_*A*_ and *n*_*B*_ as the concentration of *A* and *B*, respectively, and *X*_*A*_, *X*_*B*_, and *Y* are the autocorrelation function for *A*, the autocorrelation function for *B*, and the pair correlation function between *A* and *B*, respectively. Note that *X*_*A*_, *X*_*B*_, and *Y* are dimensionless. The general set of PDEs governing the dynamics of the MPDs based on the reactions outlined above are

$$\partial \rho_{m,m\prime }/\partial t=-\sum_{i=1}^{m}\sum_{j=1}^{m\prime }\mu \sigma (r_{i}-r_{j}^{\prime })\rho_{m,m\prime }-\sum_{i=1}^{m}\int \mu \sigma (r_{i}-r_{m\prime +1}^{\prime })\rho_{m,m\prime +1}d^{3}r_{m\prime +1}^{\prime }+k_{\text{on}}^{*}(m\prime \rho_{m+1,m\prime -1})+k_{\text{off}}(m\rho_{m-1,m\prime +1})-(\lambda +k_{\text{on}}^{*})(m\rho_{m,m\prime })-k_{\text{off}}(m\prime \rho_{m,m\prime })$$

where the parameters have been previously defined. The explicit expressions for the first five MPDPDEs are

$$\begin{array}{c} \partial \rho_{1,0}/\partial t=-(k_{\text{on}}^{*}+\lambda )\rho_{1,0}+k_{\text{off}}\rho_{0,1}-\int \mu \sigma (r_{1}-r_{1}^{\prime })\rho_{1,1}d^{3}r_{1}^{\prime }\\ \partial \rho_{0,1}/\partial t=k_{\text{on}}^{*}\rho_{1,0}-k_{\text{off}}\rho_{0,1} \end{array}$$

$$\partial \rho_{1,1}/\partial t=-(k_{\text{on}}^{*}+k_{\text{off}}+\lambda )\rho_{1,1}+k_{\text{on}}^{*}\rho_{2,0}+k_{\text{off}}\rho_{0,2}-\mu \sigma (r_{1}-r_{1}^{\prime })\rho_{1,1}-\int \mu \sigma (r_{1}-r_{2}^{\prime })\rho_{1,2}d^{3}r_{2}^{\prime }$$

$$\begin{array}{c} \partial \rho_{2,0}/\partial t=2k_{\text{off}}\rho_{1,1}-2(k_{\text{on}}^{*}+\lambda )\rho_{2,0}-\int \mu \sigma (r_{1}-r_{1}^{\prime })\rho_{2,1}d^{3}r_{1}^{\prime }-\int \mu \sigma (r_{2}-r_{1}^{\prime })\rho_{2,1}d^{3}r_{1}^{\prime }\\ \partial \rho_{0,2}/\partial t=2k_{\text{on}}^{*}\rho_{1,1}-2k_{\text{off}}\rho_{0,2} \end{array}$$

To uncouple the infinite hierarchy of these PDEs, we use Kirkwood’s approximation

$$\langle n(r_{1})n(r_{2})n(r_{3})\rangle \approx \frac{\langle n(r_{1})n(r_{2})\rangle \langle n(r_{1})n(r_{3})\rangle \langle n(r_{2})n(r_{3})\rangle }{\langle n(r_{1})\rangle \langle n(r_{2})\rangle \langle n(r_{3})\rangle }$$

which in our case leads to

$$\rho_{1,2}\approx \frac{\langle n_{A}(r_{1})n_{B}(r_{1}^{\prime })\rangle \langle n_{A}(r_{1})n_{B}(r_{2}^{\prime })\rangle \langle n_{B}(r_{1}^{\prime })n_{B}(r_{2}^{\prime })\rangle }{\langle n_{A}(r_{1})\rangle \langle n_{B}(r_{1}^{\prime })\rangle \langle n_{B}(r_{2}^{\prime })\rangle }=n_{A}n_{B}^{2}X_{B}(r_{1}^{\prime }-r_{2}^{\prime })Y(r_{1}-r_{1}^{\prime })Y(r_{1}-r_{2}^{\prime })$$

and

$$\rho_{2,1}\approx \frac{\langle n_{A}(r_{1})n_{A}(r_{2})\rangle \langle n_{A}(r_{1})n_{B}(r_{1}^{\prime })\rangle \langle n_{A}(r_{2})n_{B}(r_{1}^{\prime })\rangle }{\langle n_{A}(r_{1})\rangle \langle n_{A}(r_{2})\rangle \langle n_{B}(r_{1}^{\prime })\rangle }=n_{A}^{2}n_{B}X_{A}(r_{1}-r_{2})Y(r_{1}-r_{1}^{\prime })Y(r_{2}-r_{1}^{\prime })$$

We next express the derivatives of the first five MPDs in terms of their definitions (*n*_*A*_, *n*_*B*_, *X*_*A*_, *X*_*B*_, and *Y*) to obtain

$$\begin{array}{c} \partial \rho_{1,0}/\partial t=\partial n_{A}/\partial t\\ \partial \rho_{0,1}/\partial t=\partial n_{B}/\partial t\\ \partial \rho_{2,0}/\partial t=2n_{A}X_{A}\partial n_{A}/\partial t+n_{A}^{2}\partial X_{A}/\partial t\\ \partial \rho_{0,2}/\partial t=2n_{B}X_{B}\partial n_{B}/\partial t+n_{B}^{2}\partial X_{B}/\partial t\\ \partial \rho_{1,1}/\partial t=n_{B}Y\partial n_{A}/\partial t+n_{A}Y\partial n_{B}/\partial t+n_{A}n_{B}\partial Y/\partial t \end{array}$$

Using these derivatives together with the simplified expressions for ρ_1,2_ and ρ_2,1_ obtained using Kirkwood’s approximation, we can simplify the first five MPDPDEs as follows

$$\begin{array}{c} \partial n_{A}/\partial t=-(k_{\text{on}}^{*}+\lambda )n_{A}+k_{\text{off}}n_{B}-n_{A}n_{B}\int \mu \sigma (r\prime )Y(r\prime )d^{3}r\prime \\ \partial n_{B}/\partial t=k_{\text{on}}^{*}n_{A}-k_{\text{off}}n_{B}\\ \partial Y/\partial t=\frac{k_{\text{on}}^{*}n_{A}}{n_{B}}(X_{A}-Y)+\frac{k_{\text{off}}n_{B}}{n_{A}}(X_{B}-Y)-\mu \sigma (r)Y-n_{B}Y\int \mu \sigma (r\prime )Y(r\prime )(X_{B}(r-r\prime )-1)d^{3}r\prime \\ \partial X_{A}/\partial t=\frac{2k_{\text{off}}n_{B}}{n_{A}}(Y-X_{A})-n_{B}X_{A}\int \mu \sigma (r\prime )Y(r\prime )(Y(r\prime -r)+Y(r\prime +r)-2)d^{3}r\prime \\ \partial X_{B}/\partial t=\frac{2k_{\text{on}}^{*}n_{A}}{n_{B}}(Y-X_{B}) \end{array}$$

The initial conditions for this integral MPDPDE system are *n*_*A*_(*t* = 0) = [peptide], *n*_*B*_(*t* = 0) = 0, *X*_*A*_(*t* = 0, *r*) = 1, *X*_*B*_(*t* = 0, *r*) = 1, and *Y*(*t* = 0, *r*) = 1.

A numerical solution of this integral MPDPDE system can be obtained by noting that there are two distinct types of integrals. The first integral, appearing in the equation for *n*_*A*_, is evaluated by defining |**r**′| = *r*′ to obtain

$$\int G(r\prime \prime )d^{3}r\prime =\int_{0}^{\infty }dr\prime \int_{0}^{2\pi }d\varphi \prime \int_{0}^{\pi }d\theta \prime [{\left(r\prime \right)}^{2}\text{sin}(\theta \prime )G(r\prime )]=4\pi \int_{0}^{\infty }dr\prime [{\left(r\prime \right)}^{2}G(r\prime )]$$

The second integral, appearing in the equations for *Y* and *X*_*A*_, is evaluated by defining $q=|r-r\prime |=\sqrt{r^{2}+{\left(r\prime \right)}^{2}-2rr\prime \;\text{cos}(\theta \prime )}$, where, without loss of generality, it is assumed that $r=r\hat{z}$, so that

$$\int G(r-r\prime )d^{3}r\prime =\int_{0}^{\infty }dr\prime \int_{0}^{2\pi }d\varphi \int_{0}^{\pi }d\theta \prime [{\left(r\prime \right)}^{2}\text{sin}(\theta \prime )G(|r-r\prime |)]=\frac{2\pi }{r}\int_{0}^{\infty }dr\prime \int_{\left|r-r\prime \right|}^{\left|r+r\prime \right|}dq[qr\prime G(q)]$$

Using these integral definitions, the definition of σ(*r*), and by rescaling *n*_*A*_ and *n*_*B*_ by [peptide] and *r* by *L*, we arrive at the following nondimensional MPDPDE system

$$\begin{array}{c} \partial n_{A}/\partial t=-(p_{1}+p_{5})n_{A}+p_{2}n_{B}-4\pi {\left(3/2\pi \right)}^{3/2}p_{3}n_{A}n_{B}\int_{0}^{\infty }dr\prime [{\left(r\prime \right)}^{2}e^{-\frac{3{\left(r\prime \right)}^{2}}{2}}Y(r\prime )]\\ \partial n_{B}/\partial t=p_{1}n_{A}-p_{2}n_{B}\\ \partial Y/\partial t=\frac{p_{1}n_{A}}{n_{B}}(X_{A}-Y)+\frac{p_{2}n_{B}}{n_{A}}(X_{B}-Y)-{\left(\frac{3}{2\pi }\right)}^{3/2}p_{4}e^{-\frac{3r^{2}}{2}}Y-2\pi {\left(\frac{3}{2\pi }\right)}^{3/2}p_{3}\frac{n_{B}Y}{r}(\int_{0}^{\infty }dr\prime \int_{\left|r-r\prime \right|}^{\left|r+r\prime \right|}dq[qr\prime e^{-\frac{3{\left(r\prime \right)}^{2}}{2}}Y(r\prime )(X_{B}(q)-1)])\\ \partial X_{A}/\partial t=\frac{2p_{2}n_{B}}{n_{A}}(Y-X_{A})-4\pi {\left(\frac{3}{2\pi }\right)}^{3/2}p_{3}\frac{n_{B}X_{A}}{r}(\int_{0}^{\infty }dr\prime \int_{\left|r-r\prime \right|}^{\left|r+r\prime \right|}dq[qr\prime e^{-\frac{3{\left(r\prime \right)}^{2}}{2}}Y(r\prime )(Y(q)-1)])\\ \partial X_{B}/\partial t=\frac{2p_{1}n_{A}}{n_{B}}(Y-X_{B}) \end{array}$$

with initial conditions *n*_*A*_(*t* = 0) = 1, *n*_*B*_(*t* = 0) = 0, *X*_*A*_(*t* = 0, *r*) = 1, *X*_*B*_(*t* = 0, *r*) = 1, and *Y*(*t* = 0, *r*) = 1.

The five fitting parameters (p_1_, p_2_, p_3_, p_4_, and p_5_) are related to the five biophysical/biochemical constants as follows: p_1_ = *k*_on_ [SHP-1], p_2_ = *k*_off_, $p_{3}=k_{\text{cat}}^{*}(\text{tethered})\times$ [peptide], $p_{4}=k_{\text{cat}}^{*}(\text{tethered})/L^{3}$, and $p_{5}=k_{\text{cat}}^{*}(\text{solution})\times$[SHP-1].

The two numerical parameters are the spatial discretization (Δ*R*) and the integration upper bound (*R*_max_). We found that Δ*R* = 0.05 and *R*_max_ = 4.5 (maximum support at which the infinite integrals were truncated) introduced errors that were substantially smaller than experimental noise while maintaining the computational efficiency required for data fitting.

The MPDPDE model was fit to the experimental data using lsqcurvefit in Matlab (Mathworks). Specifically, the value of *n*_*B*_ from the MPDPDE model was fit to the experimental SPR data that were normalized to maximum binding. Each fit was repeated multiple times with different initial guesses for the five fitting parameters to make certain that the best fit was achieved (global convergence). Furthermore, we performed MCMC, using a previously published Matlab toolbox (*45*), on a subset of the experimental data to show that the five fitted parameters can be determined independently from a single SPR time course (see fig. S5 for MCMC analysis of the fit in Fig. 2A). The numerical code for solving the MPDPDE model in Matlab is provided (Supplementary Online Material). All experimental data in the present manuscript is provided as an Excel spreadsheet (Supplementary Online Material).

### Solution phosphatase assay

Purified SHP-1 was mixed with PEG12-ITIM peptide at 10 μM, in 10 mM Hepes (pH 7.4), 150 mM NaCl, 3 mM EDTA, 0.005% Surfactant P20, and 1 mM dithiothreitol (Sigma). Temperature was regulated to 10°C with a thermocycler heat block, and the production of inorganic phosphate was measured at the indicated time points using BIOMOL Green (Enzo Life Sciences).

The resulting progress curves (Fig. 1B) were fit using a standard mathematical model (*20*) based on the following reaction scheme

$$F+S^{*}\overset{k_{\text{on}}}{\underset{k_{\text{off}}}{⇌}}C\underset{⇀}{k_{\text{cat}}}F+S$$

where *F* is the phosphatase, S* is the phosphorylated peptide substrate, *S* is the unphosphorylated peptide product, and *C* is the intermediate enzyme-substrate complex. This reaction scheme corresponds to the following coupled ODEs

$$\begin{array}{c} \partial F/\partial t=-k_{\text{on}}FS*+(k_{\text{off}}+k_{\text{cat}})C\\ \partial S*/\partial t=-k_{\text{on}}FS*+k_{\text{off}}C\\ \partial C/\partial t=k_{\text{on}}FS*-(k_{\text{off}}+k_{\text{cat}})C\\ \partial S/\partial t=k_{\text{cat}}C \end{array}$$

with the following conservation equations for the enzyme and substrate,

$$\begin{array}{c} F_{T}=E+C\\ S_{T}=S*+C+S \end{array}$$

where *F*_*T*_ is the initial SHP-1 concentration and *S*_*T*_ is the initial concentration of phosphorylated substrate. A common assumption for in vitro solution-based enzyme assays is that the enzyme-substrate complex (*C*) changes on a slower time scale compared to the time scale of product formation (that is, ∂*C*/∂*t* ≈ 0). Using this quasi–steady-state approximation, which is valid when *E* ≪ *S* + *K*_m_ (*46*, *47*), we find *C* = *F*_*T*_*S*^∗^/(*K*_m_ + *S*^∗^). Using this result, together with the conservation of substrate, we arrive at a simple ODE for the production of unphosphorylated peptide

$$\frac{\partial S}{\partial t}=\frac{k_{\text{cat}}F_{T}(S_{T}-S)}{K_{m}+(S_{T}-S)}$$

where *k*_cat_ is the catalytic rate (in the unit s^−1^) and *K*_m_ is the Michaelis-Menten constant (in micromolar). The initial condition is *S*(*t* = 0) = 0.

This ODE was solved using ode45 and fit to experimental data using the function lsqcurvefit in Matlab (Mathworks). We found that a simultaneous fit of the model to all the data was sufficient to uniquely determine the three model parameters (*k*_cat_, *K*_m_, and *S*_*T*_).

## Supplementary Material

http://advances.sciencemag.org/cgi/content/full/3/3/e1601692/DC1

## SUPPLEMENTARY MATERIALS

Supplementary material for this article is available at http://advances.sciencemag.org/cgi/content/full/3/3/e1601692/DC1 fig. S1. Unprocessed SPR traces for the data in Fig. 2A showing the binding trace for the experimental flow cell (black) and the control flow cell (red). fig. S2. Point mutations to both SH2 domains of SHP-1 result in minimal binding but appreciable dephosphorylation. fig. S3. Comparison of the standard and MPDPDE model fits. fig. S4. Theoretical SPR traces generated by the MPDPDE model. fig. S5. MCMC analysis of the experimental data in Fig. 2A highlights that all five parameters can be determined independently of each other. fig. S6. Quality control of experimental data. fig. S7. Surface tethering markedly increases the rate of dephosphorylation. fig. S8. Calculation of local concentration, σ(*r*), based on two polymers a distance of *r* apart that can be approximated by worm-like chains with parameter *L*_*A*_ for the free phosphorylated peptide and *L*_*B*_ for the SHP-1–bound phosphorylated peptide. Supplementary code Supplementary data (Microsoft Excel format)
