# Supplementary material for: Biophysical assay for tethered signaling reactions reveals tether-controlled activity for the phosphatase SHP-1
Source: Sci Adv. 2017 Mar 24;3(3):e1601692. doi: 10.1126/sciadv.1601692 (PMC5365251; doi:10.1126/sciadv.1601692)
Supplement: http://advances.sciencemag.org/cgi/content/full/3/3/e1601692/DC1 [file supp_3_3_e1601692__index.html]

Science Advances | Science Advances

## Supplementary Materials

**This PDF file includes:**

- fig. S1. Unprocessed SPR traces for the data in Fig. 2A showing the binding trace for the experimental flow cell (black) and the control flow cell (red).
- fig. S2. Point mutations to both SH2 domains of SHP-1 result in minimal binding but appreciable dephosphorylation.
- fig. S3. Comparison of the standard and MPDPDE model fits.
- fig. S4. Theoretical SPR traces generated by the MPDPDE model.
- fig. S5. MCMC analysis of the experimental data in Fig. 2A highlights that all five parameters can be determined independently of each other.
- fig. S6. Quality control of experimental data.
- fig. S7. Surface tethering markedly increases the rate of dephosphorylation.
- fig. S8. Calculation of local concentration, σ(*r*), based on two polymers a distance of r apart that can be approximated by worm-like chains with parameter *LA* for the free phosphorylated peptide and *LB* for the SHP-1–bound phosphorylated peptide.

Download PDF

**Other Supplementary Material for this manuscript includes the following:**

- Supplementary code
- Supplementary data (Microsoft Excel format)

**Files in this Data Supplement:**

- Adobe PDF - 1601692\_SM.pdf
