# Supplementary material for: Biophysical assay for tethered signaling reactions reveals tether-controlled activity for the phosphatase SHP-1
Source: Sci Adv. 2017 Mar 24;3(3):e1601692. doi: 10.1126/sciadv.1601692 (PMC5365251; doi:10.1126/sciadv.1601692)
Supplement: http://advances.sciencemag.org/cgi/content/full/3/3/e1601692/DC1 [file 1601692_SM.pdf]

Science Advances

AAAS

advances.sciencemag.org/cgi/content/full/3/3/1601692/DC1

Supplementary Materials for

Biophysical assay for tethered signaling reactions reveals tether-controlled activity for the phosphatase SHP-1

Jesse Goyette, Citlali Solis Salas, Nicola Coker-Gordon, Marcus Bridge, Samuel A. Isaacson, Jun Allard, Omer Dushek

Published 24 March 2017, *Sci. Adv.* **3**, e1601692 (2017)
DOI: 10.1126/sciadv.1601692

The PDF file includes:

- fig. S1. Unprocessed SPR traces for the data in Fig. 2A showing the binding trace for the experimental flow cell (black) and the control flow cell (red).
- fig. S2. Point mutations to both SH2 domains of SHP-1 result in minimal binding but appreciable dephosphorylation.
- fig. S3. Comparison of the standard and MPDPDE model fits.
- fig. S4. Theoretical SPR traces generated by the MPDPDE model.
- fig. S5. MCMC analysis of the experimental data in Fig. 2A highlights that all five parameters can be determined independently of each other.
- fig. S6. Quality control of experimental data.
- fig. S7. Surface tethering markedly increases the rate of dephosphorylation.
- fig. S8. Calculation of local concentration, $\sigma(r)$, based on two polymers a distance of r apart that can be approximated by worm-like chains with parameter L_A for the free phosphorylated peptide and L_B for the SHP-1–bound phosphorylated peptide.

Other Supplementary Material for this manuscript includes the following:
(available at advances.sciencemag.org/cgi/content/full/3/3/e1601692/DC1)

- Supplementary code
- Supplementary data (Microsoft Excel format)

Supplementary Figures

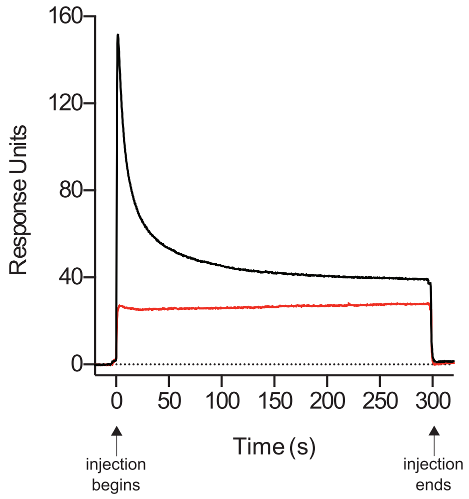

fig. S1. Unprocessed SPR traces for the data in Fig. 2A showing the binding trace for the experimental flow cell (black) and the control flow cell (red). Processed data is obtained by substracting the control flow cell from the experimental flow cell and normalising the resulting curve by the theoretical maximum SHP-1 binding (Fig. 2A, see Materials & Methods).

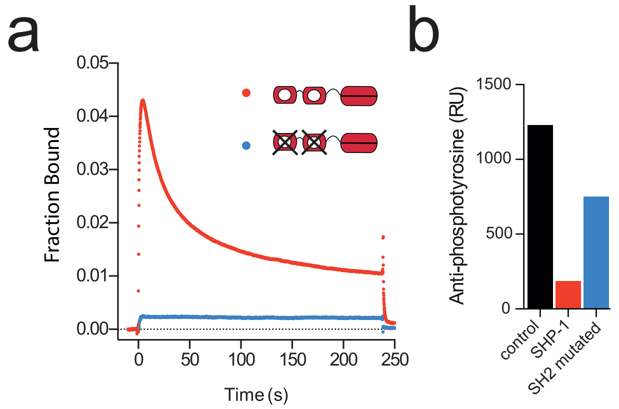

fig. S2. Point mutations to both SH2 domains of SHP-1 result in minimal binding but appreciable dephosphorylation. (**a**) Processed SPR traces of wild-type SHP-1 (red dots) and N- and C-SH2 binding null double mutant SHP-1 (blue dots) injected over flow cells with similar ITIM peptide levels (158 μM and 167 μM, respectively). (**b**) Surviving phosphotyrosines from **a** were assayed by injecting anti-phosphotyrosine antibody (clone 4G10) over a control flow cell (control; ITIM immobilised but no SHP-1 injected), or flow cells that had been injected with wild type SHP-1 (SHP-1) or N- and C-SH2 binding null double mutant SHP-1 (SH2 mutated). Results demonstrate that the double SH2 mutant could still dephosphorylate PEG28-ITIM from solution, albeit more slowly than wild-type SHP-1, and that binding directly via the catalytic domain was likely too transient to contribute significantly to the overall signal in the wild type SHP-1 SPR trace.

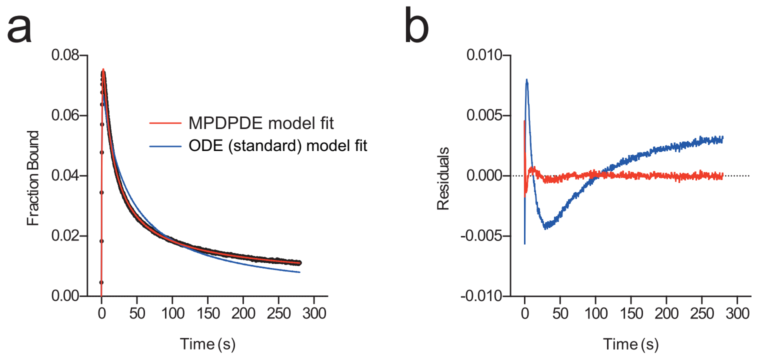

c

	MPDPDE model fit	ODE (standard) model fit
Parameter number	5	4
R^2	0.999512	0.959526
SSR	1.05 x10^{-4}	8.71 x10^{-3}
F-score	114379	
P-value	0 (to 8 digits)	

fig. S3. Comparison of the standard and MPDPDE model fits. (**a**) Example SPR trace (black dots) fit with the standard model (blue line) and the MPDPDE model (red line). (**b**) Residual trace from model fits in **a** shows clear and large systematic deviation for the standard model fit. (**c**) Goodness of fit measures (R^2 and SSR) indicate the MPDPDE model provides a superior fit to the data compared to the standard model. This is statistically supported by an F-test that produces an exceedingly small P-value for the null hypothesis that the two models fit the data equally well. The F-test requires that the two models being tested are nested, which is the case here because in the limit of large L (i.e. $p_4 \to 0$) the MPDPDE model reduces to the standard model.

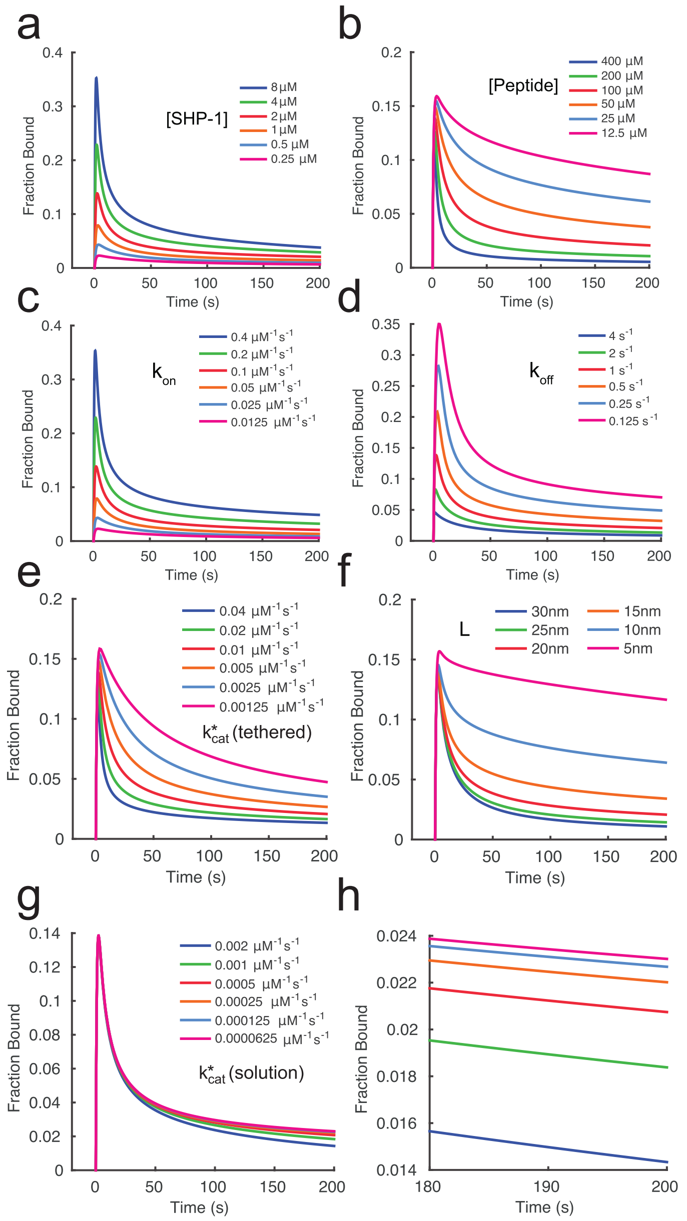

fig. S4. Theoretical SPR traces generated by the MPDPDE model. Each panel depicts the fraction of SHP-1 bound over time when varying the (**a**) SHP-1 concentration, (**b**) peptide concentration, (**c**) k_{on}, (**d**) k_{off}, (**e**) k^*_{cat} (tethered), (**f**) L, and (**g**) k^*_{cat} (solution). (**h**) An expanded view of the k^*_{cat} (solution) curves at late time points is also shown to clarify the difference between curves. Note that variation in peptide concentration changes the shape of the curve as a result of a different fraction of peptides being tethered versus solution dephosphorylated. Default parameter values are [SHP-1] = 2 μM, [peptide] = 100 μM, k_{on} = 0.1 $\mu\mathrm{M}^{-1}\mathrm{s}^{-1}$, k_{off} = 1 s^{-1}, k^*_{cat} (tethered) = 0.01 $\mu\mathrm{M}^{-1}\mathrm{s}^{-1}$, L = 20 nm, and k^*_{cat} (solution) = 0.0005 $\mu\mathrm{M}^{-1}\mathrm{s}^{-1}$.

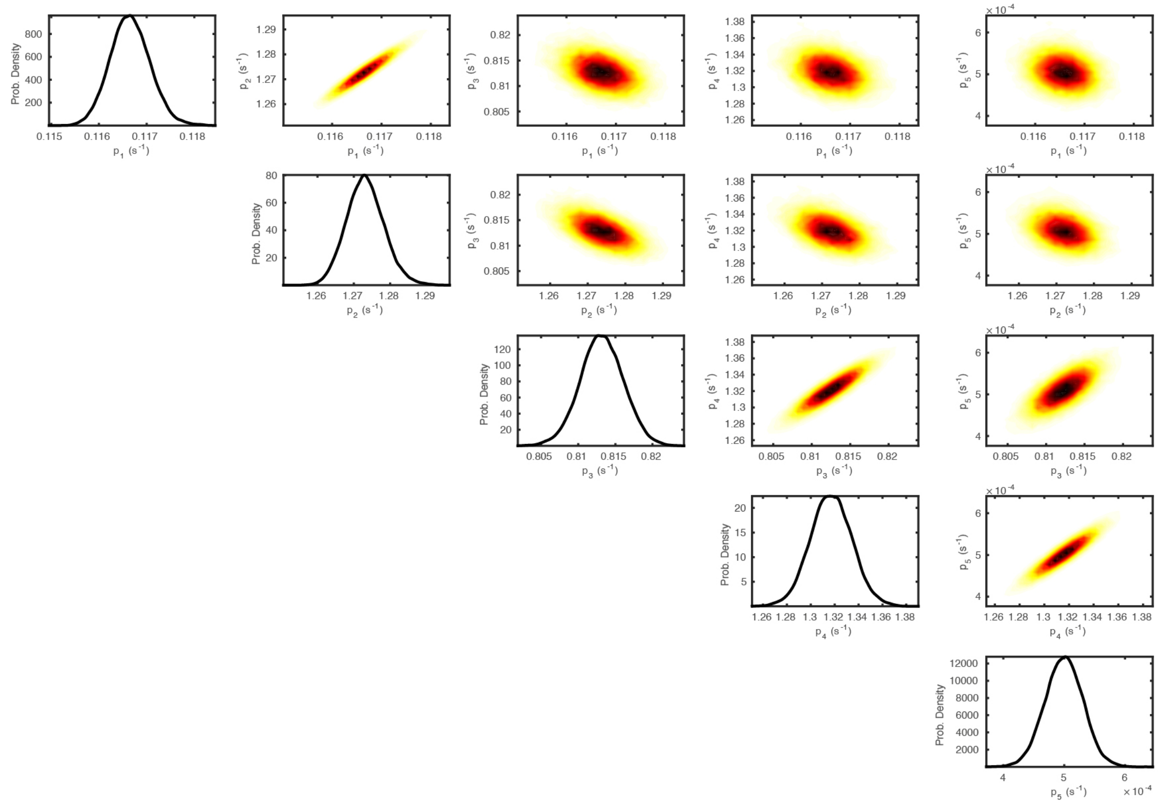

fig. S5. MCMC analysis of the experimental data in Fig. 2A highlights that all five parameters can be determined independently of each other. Panels along the diagonal show histograms of the accepted values for the five parameters revealing a small variance, indicating that parameters are determined with high accuracy. Panels off the diagonal illustrate pairwise correlations between the five parameters as heat maps, showing that parameters in the model cannot compensate for each other. See Materials & Methods for details.

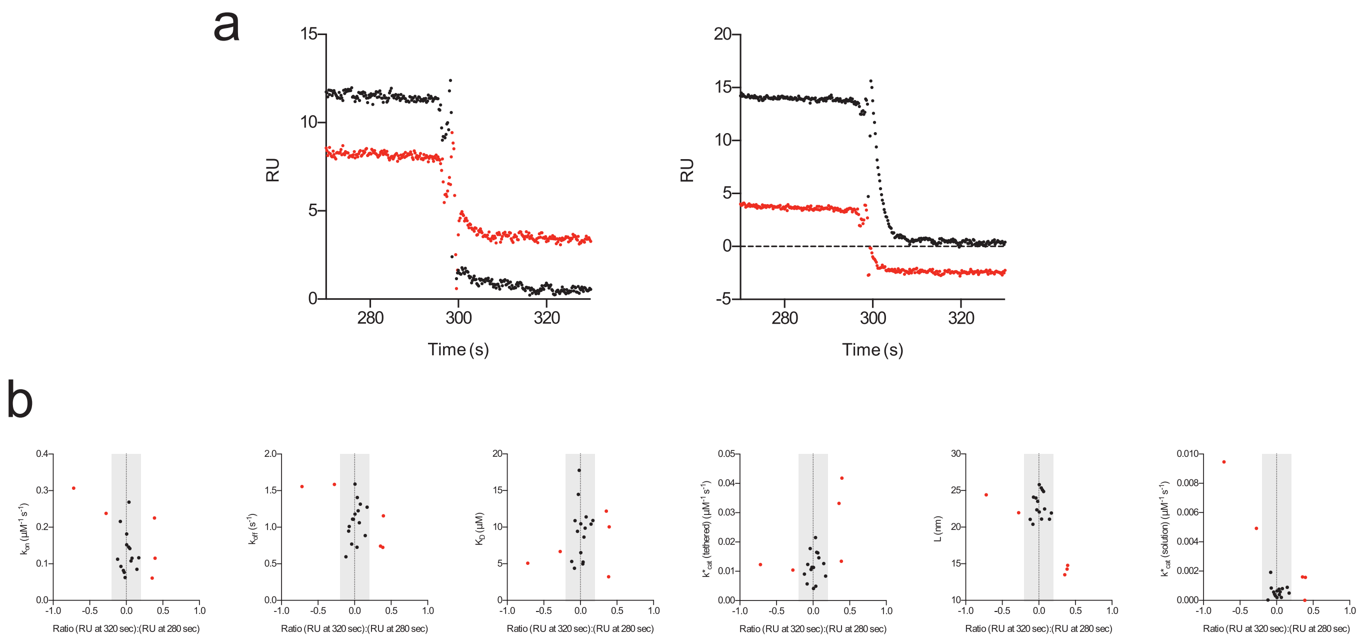

fig. S6. Quality control of experimental data. (**a**) Background subtracted but non-normalised example SPR traces of good data that returns to baseline (black) or data showing evidence of long timescale artefacts such as non-specific binding and/or differential flow cell drift (red). A characteristic trait of data affected by these artefacts is the failure to return to baseline after SHP-1 injection has completed (injection ends at 300 seconds). (**b**) Fit parameters over the ratio of RU at 320 seconds (when the SHP-1 injection has completed and the protein has dissociated) over the RU at 280 seconds (shortly before injection is complete). When the ratio is large or small, which is indicative of long timescale artefacts, we find that the fitted parameters exhibit larger variability. Particularly sensitive are L, k^*_{cat} (tethered) and k^*_{cat} (solution) whilst k_{on}, k_{off} and their ratio K_{D} are less sensitive. We chose ratio values of -0.2 and 0.2 (indicated with gray shading) as a cutoff for the exclusion of data significantly affected by long timescale artefacts (red data points).

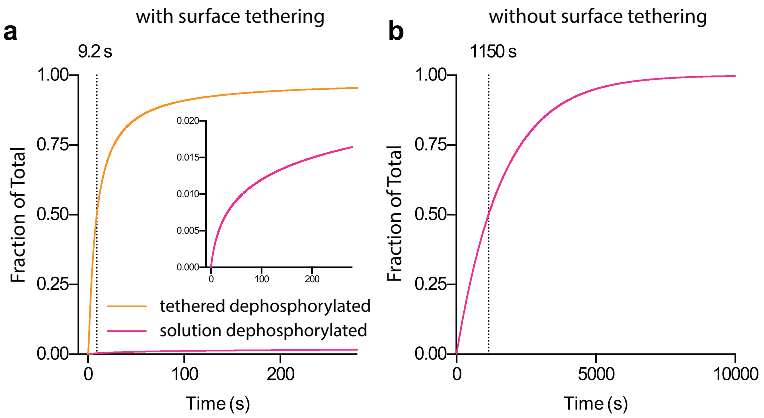

fig. S7. Surface tethering markedly increases the rate of dephosphorylation. Fraction of total phosphorylated peptide that is dephosphorylated by SHP-1 bound to the surface (surface dephosphorylated) or by SHP-1 free in solution (solution dephosphorylated) generated by the MPDPDE model in the case when (**a**) allowing surface tethering ($k_{\mathrm{on}} = 0.130\ \mu\mathrm{M}^{-1}\mathrm{s}^{-1}$) and (**b**) when preventing surface tethering ($k_{\mathrm{on}} = 0\ \mu\mathrm{M}^{-1}\mathrm{s}^{-1}$). Vertical lines indicate the time to reach 50% dephosphorylation. Parameters values: [SHP-1] = 1 μM, [Peptide] = 100 μM, $k_{\mathrm{on}} = 0.130\ \mu\mathrm{M}^{-1}\mathrm{s}^{-1}$, $k_{\mathrm{off}} = 1.08\ \mathrm{s}^{-1}$, k^*_{cat} (tethered) $= 0.0118\ \mu\mathrm{M}^{-1}\mathrm{s}^{-1}$, $L = 23.0$, and k^*_{cat} (solution) $= 0.000603\ \mu\mathrm{M}^{-1}\mathrm{s}^{-1}$ (as reported in Fig. 4b).

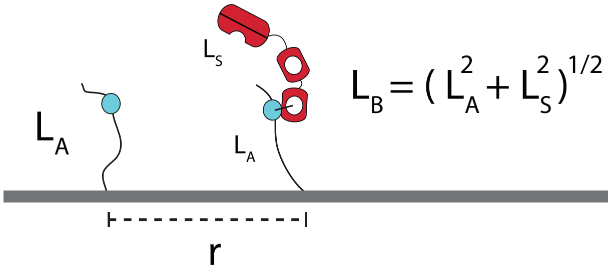

fig. S8. Calculation of local concentration, $\sigma(r)$, based on two polymers a distance of r apart that can be approximated by worm-like chains with parameter L_A for the free phosphorylated peptide and L_B for the SHP-1-bound phosphorylated peptide. Note that L_B can be decomposed into components for the phosphorylated peptide and SHP-1: $L_B^2 = L_A^2 + L_S^2$, where L_S is the worm-like-chain parameter for SHP-1. See Materials & Methods for calculation details.
